# Development and validation of the Korean version of the medication regimen complexity index

**DOI:** 10.1371/journal.pone.0216805

**Published:** 2019-05-16

**Authors:** Sunmin Lee, JunYoung Jang, Seungwon Yang, Jongsung Hahn, Kyoung Lok Min, Eun hee Jung, Kyung sun Oh, Raejung Cho, Min Jung Chang

**Affiliations:** 1 Department of Pharmaceutical Medicines and Regulatory Science, Colleges of Medicine and Pharmacy, Yonsei University, Incheon, Republic of Korea; 2 Department of Pharmacy, Inha University Hospital, Incheon, Republic of Korea; 3 Department of Pharmacy and Yonsei Institute of Pharmaceutical Sciences, College of Pharmacy, Yonsei University, Incheon, Republic of Korea; The University of Sydney School of Pharmacy, AUSTRALIA

## Abstract

The medication regimen complexity index (MRCI), originally developed in English, is a reliable and valid tool to assess the complexity of pharmacotherapy. This study aimed to validate the Korean version of MRCI (MRCI-K). A cross-cultural methodological study comprising 335 discharged patients of a tertiary hospital in Korea was conducted. The translation process included translation into Korean by two clinical pharmacists, back translation by two native speakers, and a pretest of the tool, culminating in the Korean version of MRCI-K. Reliability analysis was assessed using inter-rater and test–retest reliability with 25 randomly selected patients. Convergent and discriminant validity analyses were conducted by correlating MRCI scores with medication number, age, sex, adverse drug reaction (ADR) reports, and length of stay. The criterion validity was confirmed through evaluation by a nine-member expert panel that subjectively ranked these regimens. The reliability analysis demonstrated excellent internal consistency (Cronbach’s α = 0.977), and the intraclass correlation coefficient exceeded 0.90 for all cases. The correlation coefficient for the number of medications was 0.955 (*P* < 0.001). Weak significant correlations were observed with age and length of stay. The MRCI-K group with ADR reports scored higher (mean, 31.8) than the group without ADR reports (mean, 27.3). The expert panel’s ranking had a stronger correlation with the MRCI ranking than the medication number ranking. MRCI-K has similar reliability and validity as MRCI and is useful for analyzing therapeutic regimens with potential applications in both practice and research in Korea.

## Introduction

Taking multiple medications for chronic health problems may increase the therapeutic complexity for patients, because many factors, such as different dosage forms, diverse dosing frequencies, and, if necessary, additional directions of the regimens, contribute to the overall complexity. In addition, previous pharmacoepidemiological studies have shown that the complexities of medication regimens tend to increase with the number of hospital transfers and different regimens from hospitals.[1] Both polypharmacy and therapeutic complexity were associated with undesirable health outcomes, such as adverse reactions, hospitalization, and decreased medication adherence.[2] Studies have demonstrated that regimens with increased complexity could lead to impaired medication adherence. Thus, it was necessary to measure the complexity and investigate risk factors to improve pharmacotherapy adherence.[3] The medication regimen complexity should be defined based on the multiple characteristics of the regimens and should not be limited to the number of medications.[4] Several measures for quantifying the medication regimen complexity have been studied.[5] The medication regimen complexity index (MRCI) is a scale composed of three sections that evaluate dosage forms, dosage frequency, and additional user instructions. Scores for each section are weighted and summed to express the degree of complexity as a single number.[4]

MRCI was the most widely used scale to quantify regimen complexity and was validated in Turkey, Spain, Portugal, and Germany.[6,7] In addition, its validity and reliability were assessed as a screening tool in a population of patients with chronic obstructive pulmonary disease, diabetes, and geriatric patients.[4][8][9–11] A number of studies revealed the association between MRCI and clinical outcomes such as adherence, readmission risk, adverse drug reaction (ADR), and other clinical conditions.[9,12]^.^ As strategies for reducing medication regimen complexity were expected to improve clinical outcomes, recent studies have focused on simplifying medication regimens.[13] The MRCI has been used as a predictor for improving treatment outcome and identifying patients who would greatly benefit from pharmacy interventions.[14]

Polypharmacy, generally defined as the use of five or more medications, among the elderly was observed in Korea. The prevalence of polypharmacy was reported at 86.4%.[15] This can be a risk factor that increases medication regimen complexity. Studies have found that polypharmacy resulted in increased medication-related problems, such as adverse effects and drug interactions.[16] Until now, proven assessment tools for therapeutic complexity have not been introduced in Korea. Therefore, the Korean version of MRCI (MRCI-K) is required to assess unnecessarily complex medication, which lowers patients’ adherence. It is also essential to evaluate the degree of medication complexity to improve the effective and safe use of medications in clinical practice.

The aim of this study was to examine the validity and reliability of the original MRCI to create the MRCI-K through cross-cultural adaptation and validation.[17] The MRCI‒K would be a useful tool to assess medication regimen complexity in Korea and evaluate associations between polypharmacy and clinical outcomes.

## Materials and methods

### Study design and population setting

This was a retrospective, cross-sectional study of hospitalized patients conducted in a tertiary hospital in Incheon, Korea. The study population consisted of all individuals hospitalized in and discharged from the respiratory medicine ward from January 2016 to March 2016 who received at least one medication from the Inha University Hospital pharmacies. The patient’s diagnosis at the time of admission was checked, and the examined prescriptions contained all the medications taken due to underlying diseases including non-prescription drugs (over-the-counter [OTC] drugs). Random sample selection was performed from the total study populations.

All prescriptions and data were obtained retrospectively from electronic medical records (EMRs) and were validated by peers. Patients who were rehospitalized during the observation period were excluded. This study was approved by the institutional review board of Inha University Hospital (IRB# 2017-04-015).

### Translation and cross-cultural adaptation

The cross-cultural adaptation methodology was used to reach equivalence between the original MRCI and the Korean version of the MRCI and to maintain the validity and reliability of the index as previous studies adopted.[17] The cross-cultural adaptation process encompasses translation and back translation and consolidation by the committee to achieve semantic, idiomatic, experiential, and conceptual equivalence across cultures. During the cross-cultural adaptation process, the MRCI-K was composed of three sections like the original MRCI.

Two clinical Korean pharmacists who are fluent in English, work in a hospital as clinical pharmacists in the USA and Korea, and naive to the index at the time of the study independently translated the original MRCI. The original version of the MRCI was independently translated into Korean and reconciled after discussion of the discrepancies, and a consensual Korean version was generated. Then, two translators whose native language was English and who were not informed about the concepts of the index independently translated the consensual Korean version back to English. This English version was compared again with the original MRCI, and casual discrepancies were corrected to make a new version. The new version was also reviewed by two other Korean clinical pharmacists, who checked the comprehensibility of each question. After checking semantic, idiomatic, cultural, and conceptual equivalences, the final MRCI-K was generated. To evaluate applicability, four pharmacists who had not yet participated in the study conducted a pilot test of the MRCI-K using virtual prescriptions. If there were questions that pharmacists could not fully understand, the authors reviewed them again and modified them with more familiar expressions or additional examples.

### Reliability analysis

To estimate inter-rater reliability, MRCI reliability was evaluated with 25 individuals randomly selected from the total sample population. Six pharmacists who had not previously participated in the study independently scored the same prescriptions using the MRCI-K. Test–retest reliability was evaluated by comparing the scores obtained by the same examiner over a 2-month interval. The intraclass correlation coefficient (ICC) was used to test reliability. To interpret the ICC, we considered low reliability as ICC < 0.5, moderate reliability as 0.50 < ICC < 0.75, good reliability as 0.75 < ICC < 0.90, and excellent reliability as ICC > 0.90.[18] All statistical analyses were conducted with IBM SPSS Statistics for Windows version 24.0 (IBM Corp., Armonk, NY).

### Validity analysis

In the evaluation of validity, there were three phases to confirm the MRCI-K as a measurement of the degree of regimen complexities: convergent validity, discriminant validity, and criterion validity. First, a population descriptive analysis was conducted for the validation.

Scoring encompassed the whole medication regimen upon discharge, including self-medication, which contains prescription and non-prescription drugs (OTC drugs). Prescriptions including baseline characteristics were obtained retrospectively from EMRs. Baseline characteristics included age, sex, length of stay, types of health insurance, diagnosis upon admission, and hospital-based ADR reports. Two researchers scored and double checked each prescription. The prescriptions upon discharge contain self-medications, dosage forms, doses, dose frequencies, days prescribed, instructions, and other notes from doctors.

Construct validity was composed of both convergent and discriminant validity. To test the convergent validity, the correlation between the score of the index and the number of medications was checked using Pearson’s correlation coefficient. Discriminant validity of the MRCI-K was evaluated by the correlation between its scores and age, sex, length of stay, and ADR report using Pearson’s correlation coefficient and the Mann–Whitney test. Data analyses were conducted to determine statistically significant differences between MRCI-K and age groups, medication numbers, and lengths of stay using the Kruskal–Wallis test.

For validity evaluation, six regimens with 10-point intervals were chosen after the exclusion of points below the 5th percentile and above the 95th percentile. Similar to the original MRCI development process, concordance of MRCI-K-based rankings with expert rankings was conducted using the weighted κ agreement statistic for criterion validity. A nine-member expert panel consisting of three local pharmacists, three local internal medicine physicians, and three hospital nurses with no previous experience using the MRCI tool independently ranked regimens on the basis of their clinical opinion. The ranking by expert panels was used as the reference standard for establishing the MRCI-K criterion-related validity. To interpret the agreement level, we considered a weak agreement as 0.40 < weighted κ < 0.59, a moderate agreement as 0.60 < weighted κ < 0.79, a strong agreement as 0.80 < weighted κ < 0.90, and an almost perfect agreement as weighted κ > 0.90.[19]

## Results

### Study population

Four patients were excluded from the analysis due to prescription instruction errors, and a total of 331 patients were ultimately included for validity analysis. The baseline characteristics of the 331 patients are represented in Table 1. For the MRCI-K validation test, a total of 331 adults were included in the study, with a mean age of 68.7 years (SD 15.3 years). A total of 57.4% of the patients were male.

**Table 1:** Baseline characteristics of the patients included in the analysis (n = 331).

Characteristics		Number (%)
Age, years (mean ± SD) (range)		68.7 ± 15.3 (19–95)
Male gender		190 (57.4%)
Hospital length of stay (days)		14.2 ± 14.9
Types of health insurance		
	National health insurance	303 (91.5%)
	Medical aid	27 (8.2%)
	Others	1 (0.3%)
Diagnosis		
	Pneumonia	128 (39%)
	NSCLC	24 (7%)
	Tuberculosis	19 (6%)
	COPD	19 (6%)
	Hemoptysis	10 (3%)
	SCLC	10 (3%)
	Influenza	9 (2.7%),
	Bronchitis	6 (1.8%)
	Others	106 (31.5%)
Polypharmacy (≥5 medications)		206 (62%)
ADR reports		64 (19.3%)
Number of medications (mean ± SD) (range)		6.1 ± 3.3 (1–18)
MRCI-K score (mean ± SD) (range)		28.2 ± 14.2 (4–72)
	Section A score	2.4 ± 1.7 (1–11)
	Section B score	11.8 ± 6.0 (0.5–33)
	Section C score	14.0 ± 8.1 (2–44)

COPD, chronic obstructive pulmonary disease; MRCI-K, medication regimen complexity index Korean version; NSCLC, non-small cell lung cancer; SCLC, small cell lung cancer

On discharge, the average number of prescribed drugs was 6.1 ± 3.3, with a mean MRCI-K score of 28.2 ± 14.2. The mean score on the MRCI-K in section A was 2.4 (SD 1.7, range 1–11 points), in section B was 11.8 (SD 6, range 0.5–33 points), and in section C was 14 (SD 8.1, range 2–44 points).

### Cross-cultural adaptation

In the instruction section, we found idiomatic and experiential differences in the translation process due to different medical environment, and all translations were reconciled by reviewing these discrepancies with a pharmacist working in the USA, and they were confirmed through pretests for pharmacists. The brand names used in the original version’s instructions were changed to similar generic names in Korea, and Latin abbreviations were replaced with Korean equivalents in accordance with previous studies.[6,7,20]

In section A, the dosage forms were adapted to equivalent forms based on the Korean pharmacopoeia, with the same weight scored by the developers. For example, the formulations not used in Korea such as pessaries were translated to equivalent items with the same score based on the Korean pharmacopoeia. Metered-dosed inhaler and dry-powder inhaler items were assigned to include all formulations currently developed and administered to patient. In section C, the research panel recommended the addition of an example to the phrase “multiple units” to avoid misinterpretation; drugs that are administered as 20 mL, 2 drops, or 20 units do not score points for “multiple units at one time.”

### Reliability analysis

Random sample selection was performed from total study populations. The 52% of the samples used for reliability were diagnosed as pneumonia which was also a majority of all patients. The MRCI-K score of the 25 sample population was also similar to the scores of all patients (MRCI-K; 28.2 ± 14.2 (mean ± SD) showing MRCI-K score of 28.9 ± 13.6).

Results of the inter-rater/test–retest reliability analysis are shown in Table 2. A high correlation was observed between the scores obtained by six observers and the same evaluator after 2 months, which indicates excellent agreement between scorings and confirms the inter-rater and test–retest reliability.

**Table 2:** Inter-rater and test–retest reliability of MRCI-K.

MRCI-K	ICC (95% CI)	
	Inter-rater	Test–retest
Section A	0.915 (0.851–0.958)	0.98 (0.955–0.991)
Section B	0.987 (0.978–0.994)	0.985 (0.966–0.993)
Section C	0.823 (0.689–0.912)	0.957 (0.903–0.981)
Total	0.977 (0.96–0.989)	0.991 (0.979–0.996)

ICC, intraclass correlation coefficient; MRCI-K, medication regimen complexity index Korean version

### Validity analysis

There was a strong correlation between MRCI scores and medication numbers, with a Pearson’s correlation coefficient of 0.955 (Table 3). MRCI-K also showed a weak correlation with length of stay (0.242**, *P* < 0.001) and age (0.155**, *P* = 0.005). The two groups were statistically different from each other depending on ADR reporting (*P* = 0.007). The mean value of the group with ADR reporting (mean = 31.8) was higher than the other group without ADR reporting (mean = 27.3). There was a significant difference between three categorical medication number groups and MRCI-K (*P* = 0.001).

**Table 3:** Correlation between the Korean Medication Regimen Complexity Index (MRCI-K) scores and characteristics of the 331 participants.

		Section A	Section B	Section C	Total	P value	Correlation with MRCI‒K scores
Age^†^, years (mean ± SD)	≤20–49	2.0 ± 0.2	10.8 ± 0.7	11.4 ± 0.9	24.2 ± 1.7	0.079	0.155*
	50–69	2.4 ± 0.1	11.4 ± 0.5	13.2 ± 0.7	27.0 ± 1.3		
	≥70	2.4 ± 0.1	12.1 ± 0.4	15.0 ± 0.6	29.6 ± 1.0		
Medication number^†^ (mean ± SD)	1–4	1.8 ± 0.0	6.4 ± 0.2	14.2 ± 0.7	22.7 ± 0.8	0.001*	0.955*
	5–9	2.3 ± 0.1	13.1 ± 0.3	13.3 ± 0.6	28.7 ± 0.6		
	≥10	3.5 ± 0.3	20.3 ± 0.6	15.7 ± 1.0	39.6 ± 1.2		
Hospital length of stay^†^, week (mean ± SD)	1	2.1 ± 0.1	10.3 ± 0.5	11.3 ± 0.6	23.8 ± 1.1	0.001*	0.242*
	2	2.4 ± 0.1	11.2 ± 0.6	13.0 ± 0.7	26.6 ± 1.4		
	≥3	2.5 ± 0.1	13.5 ± 0.5	17.5 ± 0.7	33.6 ± 1.2		
Reported ADRs ‡ (mean ± SD)	Yes	2.5 ± 0.2	13.6 ± 0.7	15.6 ± 0.9	31.8 ± 1.6	0.007*	NA
Reported ADRs ‡ (mean ± SD)	No	2.3 ± 0.1	11.3 ± 0.3	13.6 ± 0.5	27.3 ± 0.8	0.007*	NA

^†^Kruskal–Wallis test

‡Mann–Whitney test

§Pearson’s correlation coefficient test

*P < 0.05

The κ values of concordance between the rankings performed by the nine experts and the rankings based on the MRCI-K were compared with the value of concordance between medication number and MRCI-K in Table 4. Concordance between nine experts’ panel rankings of patient-level medication regimen complexity with MRCI-K was almost perfect. Concordance with medication numbers was also strong, but was lower than the concordance of MRCI-K. The concordance between the two nurses was weak.

**Table 4:** Concordance of expert panel ranking with MRCI-K score and medication count rankings quadratic weighted κ (95% CI).

	Quadratic Weighted κ (95% CI)	
	MRCI-K ranking	Medication number ranking
Pharmacist 1	1 (1**‒**1)	0.83 (0.65**‒**1)
Pharmacist 2	0.83 (0.65**‒**1)	0.66 (0.34**‒**0.97)
Pharmacist 3	0.82 (0.60**‒**1)	1 (1**‒**1)
Internal medicine physician 1	1 (1**‒**1)	0.83 (0.60**‒**1)
Internal medicine physician 2	1 (1**‒**1)	0.83 (0.60**‒**1)
Internal medicine physician 3	0.83 (0.65**‒**1)	0.66 (0.34**‒**0.97)
Nurse 1	0.31 (0.03**‒**0.60)	0.31 (-0.03**‒**0.66)
Nurse 2	0.31 (-0.21**‒**0.84)	0.49 (-0.02**‒**0.99)
Nurse 3	1 (1**‒**1)	0.83 (0.60**‒**1)

MRCI**-**K, medication regimen complexity index-Korean version

## Discussion

Polypharmacy and complex drug usage could cause low adherence and incorrect drug usage, potentially resulting in insufficient efficacy and higher rates of adverse events.[21] The MRCI-K is the first validated Korean version of MRCI based on cross-cultural adaptation.[17] The validity test was conducted similarly to the original MRCI methodology and population group.[4] The scoring was conducted on all medications prescribed due to underlying disease including diagnosis one of admission.

Because MRCI is a measure for health professionals and not patient-reported, the significant need for major changes did not exist when translating from original version to Korean. When all translations including translations and back translations of the original MRCI have been reviewed and the discrepancies between instructions were identified, the original instruction of the MRCI was reflected. Unused products such as pessaries and oxygen/concentrator were translated into similar formulations semantically. As new devices like dry-powder inhaler and metered-dosed inhaler have been introduced, scoring for new devices was assigned based on the original questionnaire, that is, 3 points for dry-powder inhaler and 4 points for metered-dosed inhaler. For example, dry-powder inhaler like Genuair was assigned 3 points depending on how the device was used. In the section B, although “inhaled oxygen for 15 hours” is not usually administered in Korea, we included it in the index of the questionnaire. During pilot test using virtual prescriptions, there were discrepancies between raters caused by misinterpretations of section C and mathematical errors, like those in previous studies.[7,8] Some raters were not scored on “break and cut” and “multiple units at once” because multidose dispensing is common in the Korean clinical setting. Therefore, making the MRCI-K automated with exact examples and guideline fitted in clinical practice in Korea is necessary.

The MRCI-K showed high test–retest and inter-rater reliabilities. The convergent validation results revealed a very high correlation between MRCI-K scores and medication numbers, and the discriminant validation was confirmed by a lack of correlations between MRCI-K scores and variables not related to medications. These results were similar to the original MRCI development evaluation and other validation studies, providing evidence that the MRCI-K is a suitable tool to evaluate medication complexity.[4][6–8,20]

The MRIC-K mean scores of this study were higher than those of other studies with more medication numbers,[20,22,23] but similar to a previous validation study of the elderly.[20,22,23] The present study included self-medication, because prescriptions upon discharge contained self-medications in the hospital. Therefore, the results in this study reflected the whole medication regimen of each patient. When we compared our findings with other cohorts, it is expected that inpatients at discharge in Korea have some of the greatest medication burdens to follow a medication regimen. These results imply that it can lead to decrease medication adherence. The mean MRCI-K score was 28.2, and the mean total medication count was 6.1. In comparison, a similar MRCI score distribution (mean = 27.2) was observed in a Spanish study with 9.8 medications, showing relatively low scores considering the difference in medication numbers.[20] In another MRCI analysis of entire prescriptions, including OTC drugs, the mean score was 29.1 with 13.1 medications.[23] In addition, the most decisive factors contributing to complexity are the number of drugs and dosage frequency.[22] However, higher scores in Korea are presumably due to high section C scores. The validation results demonstrate that the scores of section C were higher than those of section B.[6–8,20,22] In the present study, typically prescribed medications contained instructions for dose delivery, time of dose, and food-related directions, even if food was irrelevant to the regimen. For these reasons, section C should be increased, especially for patients taking the prescribed medications together with different instructions. Food-related instructions lead to medication regimen complexity as a result of increasing dosing frequency when each medication requires a different time of administration. Dosing factors such as extra and food-related instructions increased medication complexity.[5] As a criterion for determining the degree of regimen complexity, all scales to measure medication complexity, including the MRCI, agree that multiple units per dose, non-oral route of administration, administration with regard to food, mixing, and measuring could increase complexity.[5] Dosing frequency has been identified as a major factor in increasing MRCI scoring.[24,25] Accordingly, studies were conducted to simplify medication regimen complexity by reducing medication frequency,[13] consolidating medication dosing times, switching dosing intervals, and reducing instructions, such as tablet spitting.[26]

In the criterion validation analysis, the results of the concordance between expert panel rankings from nurses were different from those of pharmacists and physicians (Table 4). Dispensing multiple drugs in one pack according to the usage is common in Korea. As a result, healthcare professionals may not recognize the factors that contribute to increases in medication complexity, including the increased frequency of dosing and increased splitting, crushing, and opening of tablets or capsules. Because nurses in hospitals are responsible for providing timely administration of medications to patients, those reasons may explain the inconsistency observed among healthcare professionals. A previous report by nurses also emphasized that simplifying usage through reconciling time of administration should be a major intervention to reduce medication complexity.[13] Therefore, dosing frequency could be considered a more important factor than the number of medications in environments similar to the one from our study. To reflect these, modified versions of MRCI-K should be developed.

In the correlation between MRCI-K scores and other variables, there was a weak correlation between MRCI-K and age, as well as MRCI-K and length of stay in our study. The MRCI-K scores of the group with ADR reporting were higher than those of other groups. Similar to other studies, weak correlation was found between MRCI-K and age, whereas there was no significant difference among them in further analyses.[8] Studies on validation and adherence have been performed specifically for elderly populations so far.[22] The mean age of our study was 68.7 years, and the elderly population occupied the majority of our sample population. Furthermore, length of stay was also associated with MRCI-K after hospitalization as assessed in previous studies.[1] The group with one more ADR report reviewed by a system of alerts showed significantly higher MRCI-K scores than the other group without ADR reports (mean = 31.8 and 27.3, respectively, *P* = 0.007). To our knowledge, this is the first study in Korea regarding the development of a medication complexity scoring index to quantify medication complexity with the MRCI. These results can be used in clinical practice to identify cases in which a high complexity may compromise therapeutic outcomes, including length of stay and adverse drug events, ensuring safety, and effectiveness in medication use. Furthermore, determination of an MRCI-K cutoff could be applied at the time of discharge or dispensing as a risk assessment tool to predict health outcomes like adherence.[11] As identified during criterion validation analysis, further studies will be needed to investigate a modified medication regimen complexity suitable for all patients and all medications, including environments such as multidose dispensing.

To our knowledge, this is the first study of MRCI conducted in Asia. The validation and international comparison of MRCI in Korea might increase its applicability and understanding of medication complexity in epidemiological research. Furthermore, we conducted an index analysis on all drugs taken by patients to ensure that the MRCI-K would be valid in the wide field of pharmacotherapy. The present study confirmed the validity and reliability of MRCI, which showed satisfactory psychometric properties for the measurement of regimen complexity used by all patients discharged from the respiratory ward over 3 months.

However, this study had several limitations. This study was conducted with a retrospective design at a single-center site and lacked information on disease severity. Validation analysis will be required to apply these findings in populations with diverse disease and medical care institutes.

## Conclusions

This study showed the satisfactory validity and reliability of the MRCI-K for the measurement of regimen complexity in Korea with potential applications in both clinical practice and research. Further studies are required to identify patients as a high complexity may compromise therapeutic outcomes and to evaluate the MRCI-K as a risk assessment tool to ensure safety and efficacy in medication use.
